# Regulation of fibroblast Fas expression by soluble and mechanical pro-fibrotic stimuli

**DOI:** 10.1186/s12931-018-0801-4

**Published:** 2018-05-10

**Authors:** Amos E. Dodi, Iyabode O. Ajayi, Christine Chang, Meghan Beard, Shanna L. Ashley, Steven K. Huang, Victor J. Thannickal, Daniel J. Tschumperlin, Thomas H. Sisson, Jeffrey C. Horowitz

**Affiliations:** 1 Department of Internal Medicine, Division of Pulmonary and Critical Care Medicine, University of Michigan Medical School, 6303 MSRB 3, 1150 W. Medical Center Drive, Ann Arbor, MI 48109-5642 USA; 2 Division of Pulmonary, Allergy and Critical Care Medicine, University of Alabama, Birmingham, USA; 3 Department of Physiology and Biomedical Engineering, Mayo Clinic, Rochester, MN USA

**Keywords:** Fibrosis, Myofibroblast, Apoptosis, Extracellular matrix, Lung injury, Wound-repair, CD-95

## Abstract

**Background:**

Fibroblast apoptosis is a critical component of normal repair and the acquisition of an apoptosis-resistant phenotype contributes to the pathogenesis of fibrotic repair. Fibroblasts from fibrotic lungs of humans and mice demonstrate resistance to apoptosis induced by Fas-ligand and prior studies have shown that susceptibility to apoptosis is enhanced when Fas (CD95) expression is increased in these cells. Moreover, prior work shows that Fas expression in fibrotic lung fibroblasts is reduced by epigenetic silencing of the Fas promoter. However, the mechanisms by which microenvironmental stimuli such as TGF-β1 and substrate stiffness affect fibroblast Fas expression are not well understood.

**Methods:**

Primary normal human lung fibroblasts (IMR-90) were cultured on tissue culture plastic or on polyacrylamide hydrogels with Young’s moduli to recapitulate the compliance of normal (400 Pa) or fibrotic (6400 Pa) lung tissue and treated with or without TGF-β1 (10 ng/mL) in the presence or absence of protein kinase inhibitors and/or inflammatory cytokines. Expression of Fas was assessed by quantitative real time RT-PCR, ELISA and Western blotting. Soluble Fas (sFas) was measured in conditioned media by ELISA. Apoptosis was assessed using the Cell Death Detection Kit and by Western blotting for cleaved PARP.

**Results:**

Fas expression and susceptibility to apoptosis was diminished in fibroblasts cultured on 6400 Pa substrates compared to 400 Pa substrates. TGF-β1 reduced Fas mRNA and protein in a time- and dose-dependent manner dependent on focal adhesion kinase (FAK). Surprisingly, TGF-β1 did not significantly alter cell-surface Fas expression, but did stimulate secretion of sFas. Finally, enhanced Fas expression and increased susceptibility to apoptosis was induced by combined treatment with TNF-α/IFN-γ and was not inhibited by TGF-β1.

**Conclusions:**

Soluble and matrix-mediated pro-fibrotic stimuli promote fibroblast resistance to apoptosis by decreasing Fas transcription while stimulating soluble Fas secretion. These findings suggest that distinct mechanisms regulating Fas expression in fibroblasts may serve different functions in the complex temporal and spatial evolution of normal and fibrotic wound-repair responses.

**Electronic supplementary material:**

The online version of this article (10.1186/s12931-018-0801-4) contains supplementary material, which is available to authorized users.

## Background

Fibroblast apoptosis is critical for the normal resolution of wound repair. In contrast, fibrotic repair is characterized by the accumulation of apoptosis-resistant fibroblasts and the disruption of normal tissue architecture due to excessive extracellular matrix (ECM) deposition and remodeling. Increasing recognition that enhanced fibroblast apoptosis can promote the resolution of established fibrosis in the lungs and other organs highlights the importance of understanding the complex mechanisms that regulate fibroblast survival and apoptosis during the evolution of normal and pathologic repair [1–5].

Fibroblast fate is determined by the integration of signals generated by cell-cell interactions, soluble mediators in the microenvironment, and molecular and biomechanical inputs from the ECM which converge to modulate the susceptibility of cells to apoptotic stimuli. When a potential apoptotic stimulus is encountered by a cell, the ultimate execution of the apoptotic program depends on stimulus strength, stimulus recognition, and the ability of the triggered signaling cascade to overcome an array of intrinsic inhibitors capable of blocking signal propagation [6, 7]. Thus, the apoptotic susceptibility of a cell at a given point in time represents a dynamic balance of pro- and anti-apoptotic mechanisms that are tightly regulated and can be skewed to favor either survival or apoptosis.

The tumor necrosis factor (TNF) receptor superfamily member Fas (CD95/Apo-1) serves a key function in the recognition and transduction of apoptotic signals through the extrinsic apoptosis pathway [6]. Fas ligand (FasL) binding to aggregated transmembrane Fas trimers promotes the assembly of the death-inducing signaling complex (DISC) and the downstream activation of the caspase cascade required for the execution phase of apoptosis [6, 8]. Data from humans with lung fibrosis, murine models of lung fibrosis, and cell culture systems support a role for Fas/FasL interactions in the pathogenesis of fibrosis, although the definitive mechanisms involved have not been determined [8–17]. FasL-expressing inflammatory cells have been identified in the bronchoalveolar lavage fluid and lung tissue of patients with idiopathic pulmonary fibrosis (IPF) and epithelial cells in IPF tissue were found to express Fas, suggesting that Fas/FasL interactions can promote ongoing alveolar epithelial cell death [18]. Additional studies confirmed strong epithelial cell Fas expression in IPF tissue and showed that myofibroblasts within fibroblast foci have minimal Fas expression, a finding that highlights the epithelial-fibroblast apoptosis paradox of IPF [8, 19, 20]. Furthermore, several studies have shown that fibroblasts from patients with fibrotic lung diseases have increased resistance to FasL-induced apoptosis, which would be expected in cells with decreased Fas expression [14, 15, 17, 21, 22]. Importantly, however, fibroblast susceptibility to apoptosis can be enhanced by inducing cell membrane Fas expression with exposure to inflammatory cytokines [19].

We have shown that murine lung fibrosis is associated with epigenetic suppression of the Fas promoter in lung fibroblasts and that fibroblasts isolated from IPF lung tissue have similar epigenetic modifications of the Fas promoter [13]. Others have recently shown that the resolution of bleomycin-induced fibrosis in mice correlates with the apoptotic susceptibility of fibroblasts co-cultured with FasL-expressing T-cells [7]. Although fibroblasts from fibrotic lungs express decreased levels of Fas and have increased resistance to apoptosis, the mechanisms by which pro-fibrotic stimuli regulate Fas expression in fibroblasts have not been explored. Given that TGF-β1 and matrix stiffness play key roles in activating fibroblasts and promoting fibroblast resistance to apoptosis, we sought to determine how these factors regulate Fas expression in normal lung fibroblasts.

## Methods

### Cell lines and culture

Normal primary human fetal (IMR-90) lung fibroblasts were obtained from ATCC (Manassas, VA). Cells between passages 8 and 16 were cultured in Dulbecco’s modified Eagle’s medium supplemented with 5% fetal bovine serum (FBS) to 60% confluence and growth arrested for 16–24 h prior to treatment. Fibroblasts were cultured on tissue culture plastic or on polyacrylamide hydrogel substrates with stiffness of either 400 Pa or 6400 Pa. The polyacrylamide hydrogels were prepared in the Tschumperlin laboratory and functionalized with type 1 collagen as previously described [23].

### Antibodies and reagents

Porcine TGF-β1, recombinant human tumor necrosis factor alpha (TNF-α) and recombinant human interferon gamma (IFN-γ) were from R&D Systems (Minneapolis, MN). Cycloheximide was from Sigma (St. Louis, MO). The activating anti-Fas antibody (clone CH11, designated as Fas-Ab) was from Millipore (Upstate, NY). PF573228, which directly blocks FAK kinase activity without significantly inhibiting PI3K or Rho-kinase (ROCK) was from Tocris Bioscience (Ellisville, MO) [24]. LY294002, (a PI3K inhibitor) was from Cell Signaling Technology (Danvers, MA). Rabbit monoclonal antibodies to glyceraldehyde-3-phosphate dehydrogenase (GAPDH), and poly-(ADP-ribose) polymerase (PARP) were from Cell Signaling (Danvers, MA; 1:1000 dilution used). The rabbit polyclonal antibody to Fas used for Western blots was from Novus Biologicals (Littleton, CO; 1:1000 dilution). The Cell Death Detection ELISA Kit detecting histone-associated DNA fragments was from Roche Applied Science (Indianapolis, IN). Horseradish peroxidase–conjugated secondary antibodies were from Pierce (Rockford, IL).

### Western immunoblot analysis and densitometry

Whole cell lysates were subjected to SDS-PAGE and Western blotting as previously described [24]. All Western blots were stripped and re-probed for GAPDH. Band densities were analyzed using the public domain NIH ImageJ program version 1.50i available at http://rsbweb.nih.gov/ij. The ratio of the band density for the target protein and for the corresponding GAPDH was determined and indexed such that the average expression in untreated controls was 1.0 and differences in expression represent “fold change”.

### Quantitative real-time RT-PCR

Quantitative real-time RT-PCR was performed using StepOne Plus Real Time PCR Systems by Applied Biosystems (Foster City, CA). Relative quantitation was based on the ΔΔC_T_ method. We used the human Fas cell surface death receptor, FAS gene (Hs00236330_m1) primer probe set and the housekeeping gene ACTB (Hs99999903_m1) from ThermoFisher Scientific (Ann Arbor, MI).

### Flow cytometry

Cells were collected by scraping and incubated with Fc Block (1:100) clone 24G2 (BD Pharmingen, San Diego, CA) for 15 min, then stained with propidium iodide, FITC Mouse Anti Human CD95 (Clone DX2) or FITC Mouse IgG1 Isotype Control, (Clone MOPC-21) antibodies (BD Pharmingen) for 30 min. Cells were run on a FACS Canto (BD Biosciences, Mountain View, CA) and further analyzed using the Flowjo single cell software analysis software.

### Fas ELISA

A 96-well MaxiSorp Immuno Plate (ThermoFischer Scientific, Ann Arbor, MI) was coated with 2 μg/ml Fas capture antibody (MAB144) from R&D Systems (Minneapolis, MN) and incubated overnight at 4 degrees Celsius. The coated plate was washed and blocked with casein in PBS for 1 h. After washing, samples and recombinant standards (326-FS-050 from R&D Systems, Minneapolis, MN) were loaded and incubated at room temperature for 2. After washing, the plate was incubated with 0.1 μg/ml Fas detection/biotinylated antibody (BAF 326, R&D Systems, Minneapolis, MN) for one hour and washed. Substrate development was done with peroxidase–conjugated streptavidin solution (Jackson ImmunoReserach Labs Inc., West Grove, PA) for 30 min. The plate was washed and color developed with 3, 3′, 5, 5’-Tetramethylbenzidine in 0.1 M acetate buffer with hydrogen peroxide (all from Sigma) for 20 min. Reaction was stopped with sulfuric acid. Absorbance was read at 450 nm. Data was analyzed SpectraMax M3 SofMax Pro 5 (Molecular Devices, Sunnyvale, CA).

### Cell surface immunofluorescence staining

20,000 fibroblasts per well were seeded on polystyrene glass slides (Falcon Brand) and cultured for 24 h prior to growth arrest in serum-free media for another 24 h prior to treatment with/without TGF-β1 (10 ng/ml) for 24 h. Cells were fixed (without permeabilization) in 4% paraformaldehyde for 30 min and non-specific binding was blocked with 3% bovine serum albumin for 1 h prior to sequential incubation with rabbit polyclonal anti-Fas antibody (Novus Biologicals catalog # NBP1–89034) at 1:50 dilution for 16 h (4 °C) and then a FITC-labeled secondary antibody (Invitrogen) at a 1:200 dilution for 1 h (room temperature). Nuclei were stained with DAPI. Fluorescence intensity was measured with NIH Image J and quantified as the “corrected total cell fluorescence” as previously reported [25].

### Statistical analysis

Statistical analysis was done using GraphPad Prism version 6.01. Comparisons for experiments with more than two conditions were made using one-way ANOVA with Tukey’s multiple comparisons test. Comparisons between two cohorts were done using an unpaired t-test. Data shown are the mean ± standard error of the mean. Each figure legends report the numbers of independent experimental replicates represented in each figure.

## Results

### Fibroblast susceptibility to apoptosis and expression of Fas are reduced on stiff substrates

**Fig. 1:**
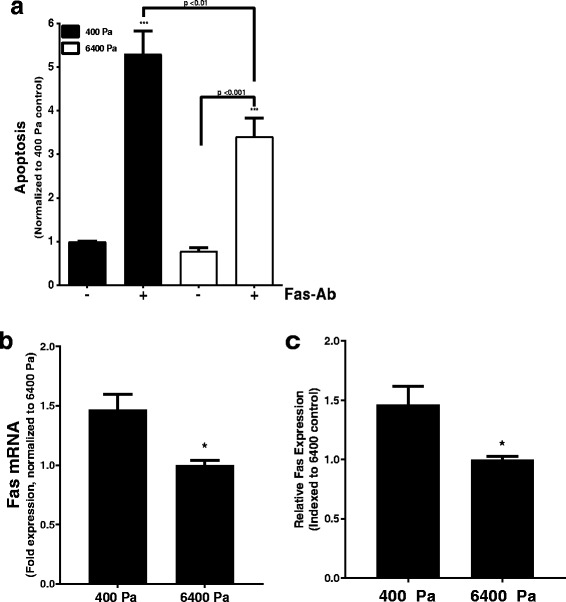
**a** Normal lung fibroblasts were cultured on polyacrylamide substrates with Young’s moduli of 400 Pa or 6400 Pa for 24 h in serum-free media prior to treatment with/without the Fas-activating antibody (Fas-Ab, 250 ng/ml) for 16 h, and apoptosis was assessed using ELISA detection of histone-associated DNA-fragments. The figure represents pooled data from five independent experiments with 3 technical replicates included in each experiment, with the data for each independent experiment normalized to 400 Pa controls to allow comparisons. *** *p* < 0.001 compared to untreated cells on 400 Pa. Comparisons were made using one-way ANOVA with Tukey’s multiple comparison test. **b** Fas mRNA in normal fibroblasts cultured for 24 h on 400 or 6400 Pa substrates was assessed by quantitative real-time RT-PCR. *n* = 3 independent experiments with two technical replicates per experiment, with data normalized to the average expression in cells on the 6400 Pa substrates. * *p* < 0.05 using unpaired T-test. **c** Fas protein in cell lysates was measured by ELISA. Data pooled from 3 independent experiments. Fas concentration was determined as pg Fas/μg protein collected and was then normalized to the average concentration of 6400 Pa substrates to allow direct comparisons between experimental replicates. * *p* < 0.05 using unpaired T-test

Biochemical and biomechanical properties of the extracellular matrix significantly impact fibroblast phenotypes including basal rates of apoptosis [26]. To determine whether substrate stiffness impacted fibroblast susceptibility to apoptosis triggered by activation of the extrinsic pathway, normal lung fibroblasts cultured on polyacrylamide hydrogels with the compliance of normal lung parenchyma (400 Pa) or on hydrogels that recapitulate the stiffness of fibrotic lung tissue (6400 Pa) were treated with/without a Fas-activating antibody (Fas-Ab, 250 ng/ml) [23]. Supporting a key role for increased matrix stiffness supporting an apoptosis-resistant fibroblast phenotype, cells cultured on the compliant (400 Pa) substrates were more susceptible to Fas-induced apoptosis than those cultured on the more rigid (6400 Pa) substrates **(**Fig. 1a**)**. We observed no significant differences in susceptibility to Fas-mediated apoptosis with further increases in substrate stiffness, including glass substrates (Additional file 1**:** Figure S1).

Others have shown that fibroblast susceptibility to Fas-ligand induced apoptosis can be regulated by enhancing cell surface Fas expression and that apoptosis-resistant fibroblasts from patients with fibrotic lung disease have decreased cell-surface Fas expression [14, 19]. To determine the effect of substrate stiffness on Fas expression, normal lung fibroblasts were seeded in serum-free media on 400 or 6400 Pa substrates for 24 h. Both Fas mRNA and protein were significantly decreased in cells on the 6400 Pa substrate **(**Fig. 1b and c**)**.

### TGF-β1 reduces Fas expression in normal lung fibroblasts on stiff substrates

**Fig. 2:**
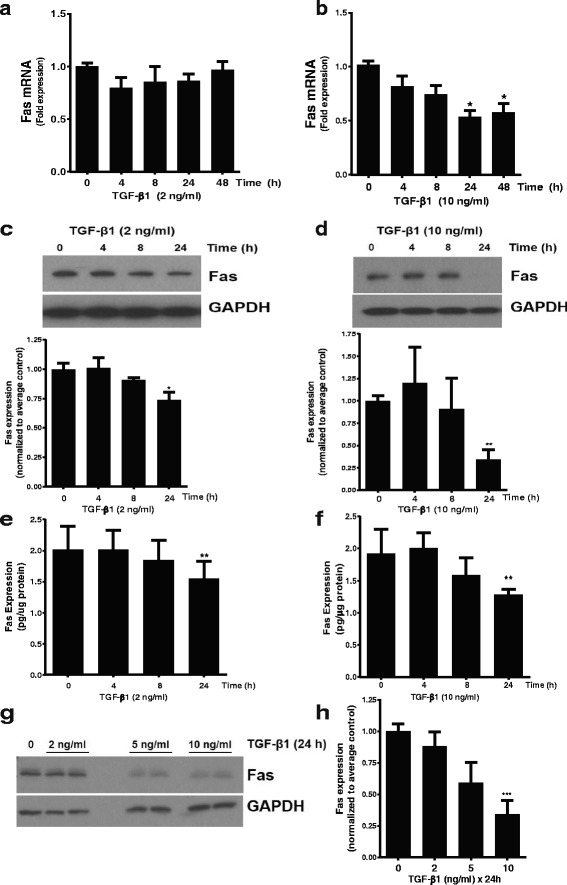
Normal lung fibroblasts were grown on tissue culture plastic in DMEM supplemented with 5% FBS to 60% confluence, serum deprived for 24 h in DMEM without serum and then treated with/without TGF-β1 at the indicated doses and durations. **a** and **b** Fas mRNA determined by quantitative real-time RT-PCR (qPCR); pooled data with an “n” of at least 7 independent replicates for each dose and time point. **c** and **d** Representative Western blots for Fas protein in whole cell lysates, with densitometric analysis. Densitometry represents data with at least 7 independent experimental replicates for each dose at the 24-h time point and 4 independent replicates at the 4- and 8-h time points. * *p* < 0.05 and ** *p* < 0.01 compared to untreated controls (One-way ANOVA with Tukey multiple comparison test). **e** and **f** Fas protein in whole cell lysates measured by ELISA. Data represent pooled results from at least 5 independent experimental replicates at the 24-h time points and 2–3 experimental replicates at the 4- and 8-h time points. ** *p* < 0.01 compared to control (One-way ANOVA with Tukey multiple comparison test). **g** Representative Western blot and (**h**) densitometry for Fas. n = at least 5 independent experimental replicates for each condition. *** *p* < 0.001 compared to untreated controls (One-way ANOVA with Tukey multiple comparison test)

TGF-β1 is a pro-fibrotic cytokine that promotes fibroblast resistance to apoptosis and is activated, in part, through non-proteolytic mechanisms that are dependent on substrate stiffness [27]. Consistent with a necessary role for increased substrate stiffness, we found that TGF-β had no significant effect on Fas expression when fibroblasts were cultured on compliant (400 Pa) polyacrylamide hydrogels (Additional file 1**:** Figure S2). To evaluate the effect of TGF-β1 on fibroblast Fas expression on stiff substrates, we measured Fas mRNA and protein in response to different doses and durations of TGF-β1 treatment **(**Fig. 2**)**. First, we found that low-dose TGF-β1 (2 ng/ml) failed to significantly impact Fas transcription over 48 h, but a higher dose (10 ng/ml) that is commonly used to study fibroblast behavior [28, 29] induced a time-dependent decrease in Fas mRNA that was significant at 24 h and persisted at 48 h **(**Fig. 2a and b**)**. Using Western blot, we observed a small but significant decline in Fas over 24 h with the 2 ng/ml dose and a more pronounced decline at 24 h following treatment with 10 ng/ml **(**Fig. 2c and d**)**. To improve the sensitivity of our assessment, we also measured total Fas in whole cell lysates (“cellular Fas”) using ELISA **(**Fig. 2e and h**)**. With this method, we did detect a statistically significant decline in Fas with 2 ng/ml which was accentuated with the 10 ng/ml dose of TGF-β1. Finally, we confirmed the dose-response relationship by assessing Fas in fibroblasts treated with 2, 5 or 10 ng/ml of TGF-β1 for 24 h via Western blot **(**Fig. 2g**)** and ELISA **(**Fig. 2h**)**. Based on the clear and consistent suppression of Fas established in these experiments, additional studies were done using TGF-β1 at a dose of 10 ng/ml.

### TGF-β1 suppression of Fas is dependent on FAK activity

**Fig. 3:**
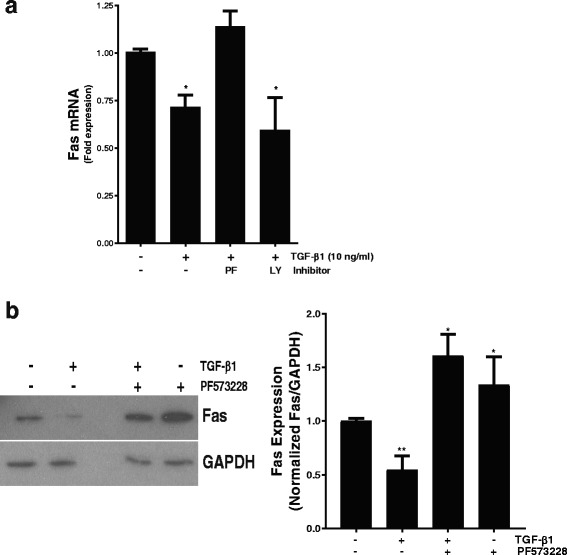
Normal lung fibroblasts were pre-treated with/without the FAK inhibitor PF573228 (10 μM) or the PI3K inhibitor LY294002 (10 μM) for one hour prior to treatment with TGF-β1 (10 ng/ml). Fas expression was then assessed by (**a**) qPCR; * p < 0.05 compared to untreated controls, *n* = 2 independent experimental replicates, (**b**) Western blot of whole cell lysates; representative blot with densitometry from three independent experimental replicates. * *p* < 0.05 and ** *p* < 0.01 compared to untreated controls

Focal Adhesion Kinase (FAK) is a non-receptor tyrosine kinase that is critical in mechanotransduction signaling, is activated by TGF-β1, mediates myofibroblast differentiation, promotes myofibroblast resistance to apoptosis, and is necessary for lung fibrogenesis [30–33]. Having found that both TGF-β1 and stiff ECM substrates suppress Fas expression in fibroblasts, we next examined the role of FAK in this process. In fibroblasts treated with the FAK inhibitor PF573228 [24], TGF-β1 failed to suppress Fas mRNA expression (Fig. 3a). In contrast, inhibition of the pro-survival PI3K/AKT signaling pathway with LY294002 had no impact on TGF-β1-mediated suppression of Fas transcription (Fig. 3a). Consistently, FAK inhibition mildly increased basal Fas expression and prevented the TGF-β-mediated reduction in Fas protein (Fig. 3b). Taken together, these experiments demonstrate that FAK activation has a critical role in TGF-β1 stimulated suppression of Fas in normal fibroblasts.

### TGF-β induces the secretion of soluble Fas

**Fig. 4:**
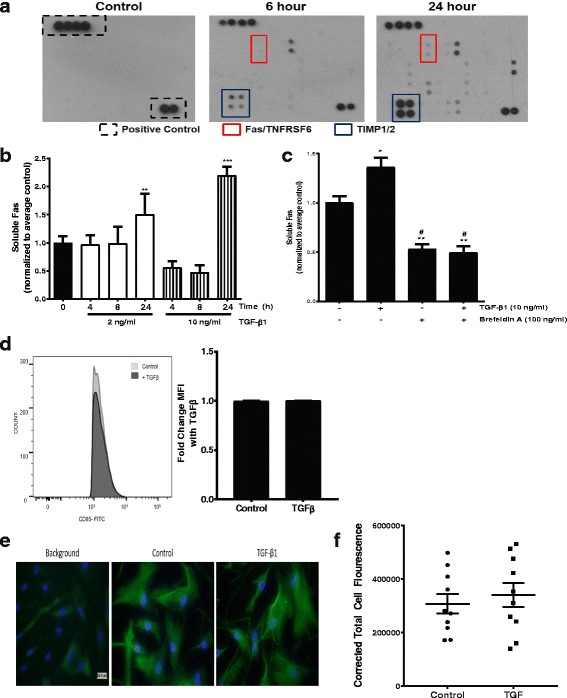
**a** Normal lung fibroblasts were treated with/without TGF-β1 (2 ng/ml) for 6 or 24 h (or were untreated controls). Proteins in the cell-culture supernatants were qualitatively assessed with the RayBio Human Cytokine Antibody Array 7.1. Shown are the assay positive controls (upper left and bottom right, black-dashed box), Fas/TNFRSF6 (red box) and TIMP1/2 (blue box). **b** sFas levels were assessed in the cell culture supernatants of normal lung fibroblasts treated with/without TGF-β1 (2 or 10 ng/ml) for the indicated time points. Data shown have been pooled from at least 3 independent experiments included with each dose- and time-point, and data have been indexed to represent the “fold-change” compared to the average of the untreated fibroblasts. ** *p* < 0.01 and *** *p* < 0.001 vs. untreated controls. **c** sFas was measured in the conditioned media from fibroblasts were treated +/− Brefeldin A (100 ng/ml) for 15–30 min then +/− TGF-β1 (10 ng/ml) for 24 h. Cell culture supernatants assessed for Fas by ELISA. Levels below assay detection were censored to the lowest detected concentration. The data shown are from 3 independent experiments. **d** Normal fibroblasts were treated with/without TGF-β1 (10 ng/ml) for 24 h. After washing, intact cells were collected by scraping and subjected to flow cytometry for Fas. A representative histogram is shown (left) along with the mean fluorescence intensity of untreated and TGF-β1 treated cells (right). *n* = 3 independent experiments. The gating strategy used is shown in Supplemental Data Fig. 3. **e** Cell-surface Fas immunofluorescence staining in normal lung fibroblasts treated with or without TGF-β1 (10 ng/ml) for 24 h. **f** Fluorescence was quantified in 10 individual cells per condition (5 cells from each of two independent experimental replicates)

Receptor expression represents the balance of protein synthesis and degradation. However, Fas is a member of the TNF-alpha superfamily, and studies have shown that these receptors, including Fas itself, can be secreted from cells as a biologically active soluble receptor [34]. Moreover, increased concentrations of soluble Fas (sFas) have been reported in the cell culture supernatants of fibrotic lung fibroblasts compared to normal lung fibroblasts [14]. In concert with proteins known to be secreted by fibroblasts, such as TIMP1 and TIMP2, we identified sFas in cell culture supernatants from normal lung fibroblasts treated with TGF-β1 for 24 h (Fig. 4a). Using ELISA, we confirmed that TGF-β1 treatment led to a significant increase in sFas in fibroblast conditioned media between 8 and 24 h, and that there was a dose-response effect to TGF-β1 (Fig. 4b). To determine if the sFas in conditioned media represented the consequence of shedding/cleavage of cell-surface receptors or if it was due to the secretion of existing cytoplasmic Fas, we pre-treated cells for one hour with Brefeldin-A, which inhibits protein transport from the endoplasmic reticulum to the Golgi and thereby blocks the protein secretory pathway. Supporting a role for the secretory pathway, TGF-β1 failed to increase the sFas in the cell culture supernatants when cells were treated with Brefeldin-A (Fig. 4c). Consistent with secretion of Fas as the primary mechanism underlying decreased fibroblast Fas, we used flow-cytometry to demonstrate that TGF-β1 did not significantly alter cell-surface Fas expression at 24 h (Fig. 4d). Similarly, there was no significant difference observed in cell-surface Fas expression when assessed by immunofluorescence staining (Fig. 4e and f).

### Augmented fibroblast susceptibility to apoptosis induced by treatment with IFN-γ and TNF-α is maintained despite treatment with TGF-β1

**Fig. 5:**
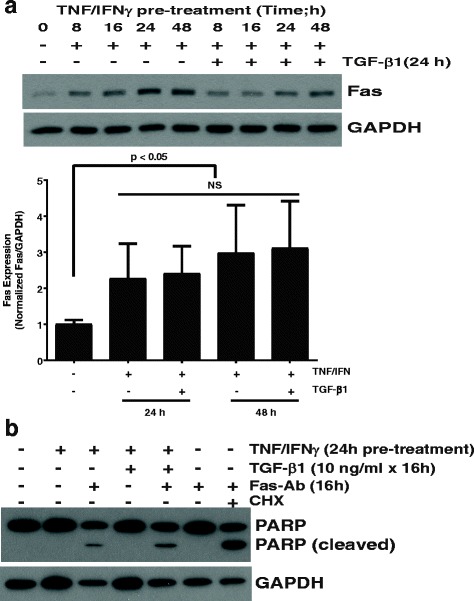
**a** Normal lung fibroblasts in serum-free media were treated with the combination of TNF-α (10 ng/ml) and IFN-γ (50 U/ml) for the indicated times up to 48 h. For the groups receiving inflammatory cytokines for 6–24 h, at the end of the exposure cells were washed with PBS and replaced in serum free media. After 48 h, the cells were washed again with PBS and placed in serum free media with/without TGF-β1 (10 ng/ml) for another 24 h. Whole cell lysates were then assessed for Fas expression. Additional experiments were done to confirm the effects at 24 and 48 h, and the densitometry represents three independent experiments at those time points. **b** Normal lung fibroblasts were treated with/without TNF-α (10 ng/ml) and IFN-γ (50 U/ml) for 24 h, washed with PBS and then exposed to Fas-activating antibody (Fas-Ab; 250 ng/ml) with/without TGF-β1 (10 ng/ml) for 16 h. Whole cell lysates were assessed for PARP. Lane 7 is from control fibroblasts treated with the Fas-activating antibody in combination with cycloheximide (CHX; 500 ng/ml) as a positive control for apoptosis

Fibroblast susceptibility to Fas-induced apoptosis is enhanced by increased cell surface Fas expression induced by combined treatment with TNF-α and IFN-γ [15, 19]. Prior studies from our laboratory have shown that TGF-β1 reduces fibroblast susceptibility to Fas-mediated apoptosis through activation of pro-survival protein kinase signaling pathways and up-regulation of intracellular inhibitors of apoptosis [4, 21, 35]. Having now shown that TGF-β also reduces fibroblast expression of Fas we sought to determine whether the anti-apoptotic effects of TGF-β1 would counter the effects of TNF-α and IFN-γ and reverse the enhanced sensitivity to apoptosis sensitivity afforded by these inflammatory cytokines. To address this, we treated normal lung fibroblasts with the combination of TNF-α and IFN-γ for between 0 and 48 h (cells treated for less than 48 h were washed with PBS and maintained in serum-free media for the remaining time until completion of the 48-h period). After 48 h, the cells were washed and treated with or without TGF-β1 for an additional 24 h, so that all of the cells were in culture for the 72-h duration of the experiment. Consistent with prior studies, fibroblasts exposed to the inflammatory cytokines for at least 8 h showed enhanced Fas expression which peaked at 24 h and persisted in the absence of TGF-β1 for the entire 72-h duration of the experiment **(**Fig. 5a) [15]. Interestingly, the cells treated with the inflammatory cytokines for 24 or 48 h followed by TGF-β1 for 24 h had no significant decline in cellular Fas. Next, we treated fibroblasts with the combination of inflammatory cytokines for 24 h prior to exposure to Fas-activating antibody alone or in combination with TGF-β1 and assessed apoptosis by Western blot for identification of cleaved PARP. The combination of cytokines alone was not sufficient to induce apoptosis, but apoptosis was apparent in cells treated with the combination of cytokines and Fas-activating antibody. Consistent with cellular Fas-expression impacting susceptibility to apoptosis, TGF-β1 failed to decrease the susceptibility to apoptosis in the cells that had been pre-treated with inflammatory cytokines **(**Fig. 5b**)**. Collectively, these findings indicate that TGF-β1 does not antagonize the increase in cell surface Fas induced by inflammatory cytokines. Moreover, the increased susceptibility to Fas-induced apoptosis afforded by treatment with the inflammatory cytokines is not altered by TGF-β1, suggesting that increasing cell-surface expression of Fas is sufficient to overcome the non-Fas mediated anti-apoptotic mechanisms induced by TGF-β1.

## Discussion

Fibroblast apoptosis heralds the resolution phase of normal repair while fibroblast resistance to apoptosis contributes to the progressive scar formation that characterizes fibrosis in the lungs and other organs. In recent years, a number of mechanisms involving inhibitor of apoptosis family proteins, BCL-2 family proteins, pro-survival protein kinase activation and cell-matrix interactions have been shown to regulate fibroblast susceptibility to apoptosis and accumulating studies demonstrate the feasibility of targeting fibroblast survival as a treatment strategy for fibrotic disease in the lung [1, 4, 21, 24, 36, 37]. Additionally, several studies now indicate that regulation of Fas itself may contribute to fibroblast acquisition of an apoptosis-resistant phenotype, although our understanding of the underlying mechanisms continues to evolve [7, 16, 20, 38]. In the current study, we have shown that pro-fibrotic soluble and mechanical stimuli suppress fibroblast expression of Fas through both transcriptional and post-transcriptional mechanisms, that decreased Fas correlates with resistance to apoptosis, and that enhanced apoptosis susceptibility in response to inflammatory cytokines is not diminished by TGF-β1 treatment.

We previously reported that fibroblasts from the lungs of mice with established fibrosis had increased resistance to apoptosis in association with decreased Fas mRNA, decreased cellular Fas and decreased membrane-bound Fas expression [13]. Moreover, we identified similar epigenetic modifications in the Fas promoter of fibroblasts from bleomycin-treated mice and from IPF lung tissue [13]. Consistently, several studies have reported that Fas is minimally expressed by myofibroblasts within the fibroblastic foci of IPF lung tissue [18–20]. Moreover, promoting expression of cell-surface Fas by treating normal or IPF fibroblasts with inflammatory cytokines is sufficient to enhance the susceptibility of these cells to apoptosis [19]. The mechanisms by which TGF-β1 and matrix stiffness impact fibroblast Fas expression, which may precede the epigenetic suppression in the earlier phases of wound repair and fibrosis, have not been elucidated.

Here, we found that Fas expression in normal lung fibroblasts was regulated by substrate stiffness and by the pro-fibrotic mediator TGF-β1. Lung fibrosis, both in humans and in mice, is associated with increased parenchymal stiffness which is not only thought to be the consequence of extracellular matrix deposition and cross-linking, but is likely to contribute to the persistence of fibrosis by directly supporting myofibroblast differentiation, matrix production and survival [23, 39–41]. Prior studies have reported that increased substrate stiffness is associated with diminished basal rates of fibroblast apoptosis. In this study, we show that substrates that recapitulate the stiffness of fibrotic lung tissue promote fibroblast resistance to FasL-induced apoptosis, and that the stiff matrix substrate is sufficient to reduce fibroblast expression of Fas.

The mechanisms by which stiff ECM substrates affect fibroblast phenotypes are likely to involve direct activation of mechanotransduction signaling pathways and accentuated liberation of active TGF-β1 from its inactive/latent form [26, 27]. Studies from our lab and others have shown a critical role for Focal Adhesion Kinase (FAK) in the regulation of myofibroblast phenotype in vitro and lung fibrosis in vivo [30–32, 42]. Accordingly, we anticipated the key role for FAK in TGF-β1 suppression of Fas transcription. However, we were surprised to find that within the time course of our experiments, decreased Fas transcription did not significantly impact expression of Fas on the cell surface. The absence of change in cell-surface Fas expression is also consistent with the failure of TGF-β1 to diminish the increased fibroblast susceptibility to apoptosis conferred by treatment with the combination of TNF-α/IFN-γ, which increase cell surface Fas expression [19].

The primary finding that TGF-β1 decreases Fas expression in fibroblasts is consistent with a recent report showing that TGF-β1 mediated downregulation of miR29c promotes suppression of Fas and increases fibroblast resistance to apoptosis [38]. However, our findings with regards to the effects of TGF-β1 on cell surface Fas expression differ from that study, which showed a relatively small (approximately 20%) reduction. The reasons for this discordance are not readily apparent, although we suspect that these small differences may reflect experimental variability due to the fibroblast cell line used and culture conditions. Allowing for this, we can conclude that the short-term effects of TGF-β1 on cell surface Fas expression are, at most, mild. Notably, prior studies have reported that fibrotic lung fibroblasts from patients with IPF and from mice following intratracheal bleomycin have decreased cell surface Fas when compared to normal lung fibroblasts [13, 14]. When taken in context with our data demonstrating that TGF-β1 decreases fibroblast transcription of Fas, we speculate over longer durations the decreased Fas transcription does lead to decreased surface receptor expression that is not evident within the duration of our experimental design. Further, it is possible that the effects of of TGF-β1 and matrix stiffness become amplified when epigenetic modifications have already suppressed Fas expression.

We found that short-term treatment of normal fibroblasts with TGF-β1 promoted the release of sFas into the cell culture media. This was prevented by treatment of fibroblasts with Brefeldin-A, suggesting that the sFas in the cell culture media was due to active secretion of protein, and not the result shedding or cleavage of the cell-surface protein. Prior studies have shown that fibroblasts from patients with lung fibrosis release increased amounts of sFas into their conditioned media [14]. Additionally, increased levels of sFas have been identified in the bronchoalveolar lavage fluid of patients with lung fibrosis [9]. The mechanistic role of sFas in the pathogenesis of lung fibrosis has not been evaluated, but studies have shown that sFas can function directly as an inhibitor of apoptosis [34, 43]. Accordingly, we speculate that TGF-β1-mediated secretion of sFas may function to allow fibroblasts to evade inflammatory cell-mediated apoptosis in the early phases of wound repair [7, 44]. We do note, however, that in our cell culture model, secretion of sFas clearly did not diminish fibroblast susceptibility to apoptosis when cell-surface Fas expression was increased by treatment with TNF-α/IFN-γ. This lack of an anti-apoptotic effect may reflect the dilution of sFas which would be expected to distribute evenly within the cell culture media, thereby reducing the concentration of the soluble receptor at the cell surface. In this cell culture model, the concentrations of Fas-Ab used to induce apoptosis have been optimized to overcome any potential dilutional effect, such that the relative ratio of sFas secreted by cells is insufficient to neutralize the Fas-Ab in the system.

Collectively, our studies and the existing literature suggest a hierarchical hypothesis whereby different mechanisms regulate fibroblast Fas expression during the evolution of wound-repair and these changes may contribute to either homeostatic repair or pathologic fibrosis depending on context. We speculate that as an early response to injury, resident fibroblasts exposed to a normally compliant substrate secrete sFas in response to TGF-β1, allowing the fibroblasts to evade apoptosis due to FasL expressed on neighboring fibroblasts and inflammatory cells [44]. As the early repair-response progresses, FAK-dependent transcriptional suppression due to a stiffening ECM substrate and continued TGF-β1 exposure leads to decreased cell surface Fas expression and decreased susceptibility to apoptosis, a potentially adaptive response that permits activated fibroblasts to persist in an environment in which apoptotic stimuli are abundant. Exposure to accumulating inflammatory cytokines then enhances cell surface Fas and enhances fibroblast susceptibility to apoptosis in the context of homeostatic repair. If, however, the temporal and spatial repair response becomes dysregulated, as may occur in the context of continued or recurrent injury or an aberrant inflammatory response, epigenetic modifications of the Fas promoter may “stabilize” an anti-apoptotic phenotype and contribute to the aberrant repair response recognized as fibrosis [13].

## Conclusions

Fibroblast susceptibility to apoptosis is key to normal and pathologic wound repair. Soluble and matrix-mediated pro-fibrotic stimuli promote fibroblast resistance to apoptosis and decreased Fas transcription while stimulating soluble Fas secretion. However, cell-surface Fas expression is not significantly altered by TGF-β1 treatment and TGF-β1 does not inhibit the enhanced fibroblast susceptibility to apoptosis induced by inflammatory cytokines. Collectively these data indicate that fibroblast expression of Fas encompasses complex temporal and compartmental regulation that may serve a variety of functions during the evolution of injury-repair responses.

## Additional file

Additional file 1: **Figure S1.** Apoptosis was assessed in normal human lung fibroblasts (IMR-90) cultured on polyacrylamide hydrogels with stiffness ranging from 400 Pa to 25,600 Pa or on glass slides for 24 h in serum-free media prior to treatment with/without a Fas-activating antibody (Fas-Ab, 250 ng/ml) for 16 h. **Figure S2.** Normal human lung fibroblasts (IMR-90) were cultured on compliant, 400 Pa polyacrylamide hydrogel substrates and treated with/without TGF-β1 (2 ng/ml) for 24 h and Fas expression was assessed. **Figure S3.** Flow cytometry analyses. (DOCX 465 kb)

## Abbreviations

ECM: Extracellular matrix
FAK: Focal adhesion kinase
FasL: Fas ligand
IFN-γ: Interferon gamma
IPF: Idiopathic pulmonary fibrosis
PARP: Poly (ADP-ribose) polymerase
PI3K: Phosphoinositide 3-kinase
sFas: Soluble Fas
TGF-β1: Transforming growth factor beta-1
TNF-α: Tumor necrosis factor alpha
