# Supplementary material for: Regulation of fibroblast Fas expression by soluble and mechanical pro-fibrotic stimuli
Source: Respir Res. 2018 May 10;19:91. doi: 10.1186/s12931-018-0801-4 (PMC5946418; doi:10.1186/s12931-018-0801-4)

**Figure S1:**

Normal human lung fibroblasts (IMR-90) were cultured on polyacrylamide hydrogels with stiffness ranging from 400 Pa to 25,600 Pa or on glass slides for 24 hours in serum-free media prior to treatment with/without a Fas-activating antibody (Fas-Ab, 250 ng/ml) for 16 hours. Apoptosis was assessed by ELISA detection of histone-associated DNA-fragments. Data are pooled from 4 independent experiments and each “stiffness” variable was included in at least 3 of the 4 experimental replicates (such that the total “n” for each condition is either 3 or 4). To allow comparisons, data for each experimental replicate have been normalized so that the extent of apoptosis in untreated fibroblasts on glass substrates is equal to 1.

**Figure S2:**

Normal human lung fibroblasts (IMR-90) were cultured on compliant, 400 Pa polyacrylamide hydrogel substrates and treated with/without TGF-b1 (2 ng/ml) for 24 hours. Fas expression was assessed by ELISA. Data are pooled from three independent experiments. For each experiment, the Fas concentration (ug/mg total protein) has been normalized to the untreated controls.

**Figure S3:**

Flow cytometry analyses: (Left most panels) cells were gated on side scatter vs. forward scatter as live cells gate. Cells that were in the live cell gate were further assessed for doublet discrimination (FSC_H vs. FSC-A). Gates show percentages of cells present. Propidium iodide was used a viable cell marker, so we then gated on PI negative cells (SSC-A vs. PI). From this gate we assessed PI negative vs. CD95-FITC positive cells to identify our population of interest (PI negative, CD95-FITC positive). Shown here is the isotype-control demonstrating background positive staining of 0.653% of untreated cells.


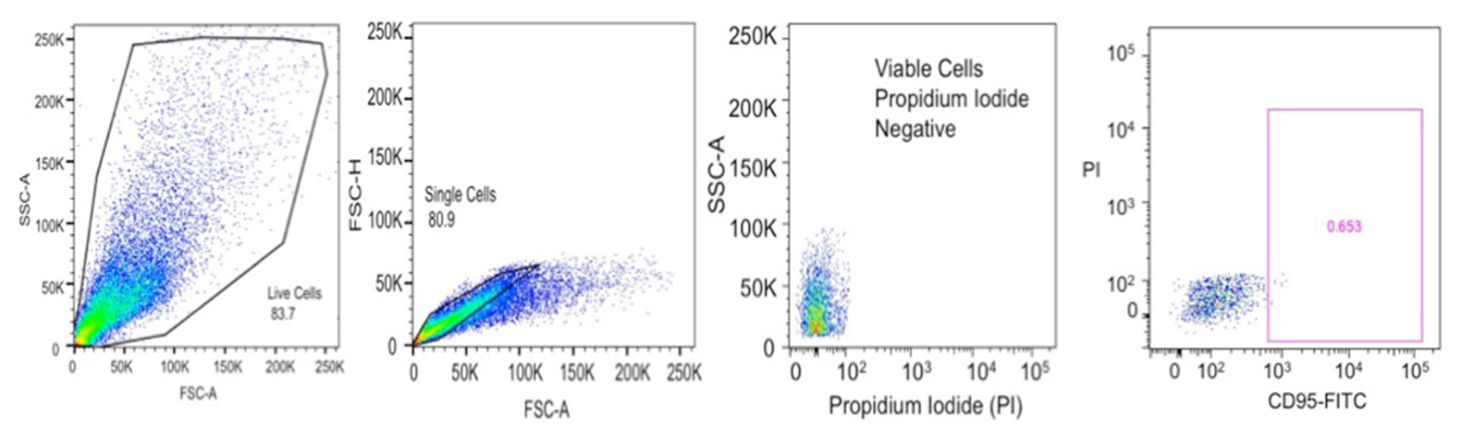

Supplement: Supplementary file 1 — Figure S1. Apoptosis was assessed in normal human lung fibroblasts (IMR-90) cultured on polyacrylamide hydrogels with stiffness ranging from 400 Pa to 25,600 Pa or on glass slides for 24 h in serum-free media prior to treatment with/without a Fas-activating antibody (Fas-Ab, 250 ng/ml) for 16 h. Figure S2. Normal human lung fibroblasts (IMR-90) were cultured on compliant, 400 Pa polyacrylamide hydrogel substrates and treated with/without TGF-β1 (2 ng/ml) for 24 h and Fas expression was assessed. Figure S3. Flow cytometry analyses. (DOCX 465 kb) [file 12931_2018_801_MOESM1_ESM.docx]
